# Incidence of first attempt peripheral intravenous cannulation failure and its predictors among children admitted to Debre Tabor Referral Hospital, Northwest Ethiopia: institution based cross-sectional clinical study

**DOI:** 10.4314/ahs.v22i4.72

**Published:** 2022-12

**Authors:** Tigabu Munye Aytenew, Demeke Mesfin Belay, Wubet Alebachew Bayih, Binyam Minuye Birhane, Abebaw Yeshambel Alemu

**Affiliations:** 1 Department of Comprehensive Nursing, College of Health Science, Debre Tabor University, Debre Tabor, Ethiopia; 2 Department of Pediatrics and Neonatal Nursing, College of Health Science, Debre Tabor University, Debre Tabor, Ethiopia

**Keywords:** Peripheral IV cannulation, predictors, cross-sectional study

## Abstract

**Background:**

When the first piercing is failed to function, repeated puncturing imposes pain, complications, and delays the timeliness of pediatric care. In spite of the above challenges, incidence and predictors of first attempt peripheral intravenous cannulation failure are under-investigated in the study area and the nation at large.

**Objective:**

This study aimed to determine the incidence of first attempt peripheral intravenous cannulation failure and its predictors among children.

**Methods:**

Institution-based cross-sectional study design was conducted, and a total of 422 children were included in the study. The study participants were selected using a simple random sampling technique. The data were collected by direct observation and interviewer-administered questionnaire. Stata version 14 was used for analysis, and finally, the association was declared using AOR at a 95% confidence level at p≤0.05.

**Results:**

The incidence of first attempt peripheral intravenous cannulation failure rate was found to be 34.83% (132). Besides, self-payment funding, vein visibility with a tourniquet, forearm site, vein scope use, and child age of 24–59 months old were significantly associated with first attempt peripheral intravenous cannulation failure.

**Conclusion:**

Generally, self-payment funding, vein visibility with a tourniquet, forearm site, vein scope use, and child age of 24–59 months old were independent predictors of first attempt peripheral intravenous cannulation failure.

## Background

Peripheral intravenous cannulation is a procedure performed for Intravenous (IV) fluid administration, medication, and/or blood and blood products. IV fluid therapy is performed to restore body fluid, electrolytes and maintain body homeostasis, when oral intake is inadequate or impossible 1. Similarly, a vein is the preferred route of drug administration when a rapid response of drugs is desired. Besides, veins can be punctured for phlebotomy and blood sample collection 2. Distal venous cannulation is a common and routine task of pediatric nursing care. Even though routinely and frequently performed, the failure rate on the first attempt, time to success, and predictors of failure vary across countries 3. In Netherlands, 23% of admissions in the operating room and 19% of children visiting outpatient were facing the first attempt IV cannulation failure 7. Similarly, a study conducted at Bhutan hospital showed that 36% of children faced the first attempt peripheral intravenous cannulation failure 9. Nonetheless, the investigators' found data were limited regarding the first attempt IV cannulation failure rate in the area and the country at large.

Repeated IV insertion because of the first attempt peripheral IV cannulation failure is challenging for caregivers, painful for parents and children, and poses complications for the baby. At two University hospitals of Neonatal Intensive Care Units (NICU) in Netherlands, infiltration as a complication was detected in 67% of admissions 4. The overall complications of IV cannulation failure are infiltration (37%), and phlebitis (5.1%) [5]. Peripheral IV cannulation via venipuncture is also the main source of procedure- related pain, anxiety and distress among children 6.

Additionally, time lapsed due to the first attempt peripheral intravenous cannulation failure might delay paediatric care. Vein puncture was unsuccessful until 3 minutes in a cohort study of Dutch hospital 7. Studies conducted at two university hospitals in US reported that patient factors (age and nutritional status), clinician factors (experience, and perception) and equipment and procedure factors (size of cannula, and light sources) were predictors of difficult peripheral venous catheterization at first trial 3, 7–9.

Factors that predict the first attempt peripheral intravenous cannulation failure were not well explained in the literature in the study area. Knowing such factors may provide support for advancing changes and innovations in the health care system, so as to obtain results that are safer and more valuable for the children. Thus, this study aimed to determine the incidence of the first attempt peripheral IV cannulation failure and identify its predictor variables.

## General objective

To determine the incidence of the first attempt peripheral intravenous cannulation failure and identify its predictors among children admitted to Debre Tabor referral hospital, Northwest Ethiopia,2020.

## Specific objectives

To determine the incidence of the first attempt peripheral intravenous cannulation failure among children admitted to Debre Tabor referral hospital, Northwest Ethiopia, 2020.

To identify the predictors of the first attempt peripheral intravenous cannulation failure among children admitted to Debre Tabor referral hospital, Northwest Ethiopia, 2020.

## Study design, Area and period

Institution-based cross-sectional study design was carried out among children admitted to Debre Tabor referral hospital, Northwest Ethiopia from October 2019 to January 2020.

### Study population

All children on whom peripheral IV cannulation was attempted from October 2019-January 2020 at Debre Tabor referral hospital.

### Inclusion and Exclusion Criteria

All children who were eligible for peripheral intravenous cannulation for any reason at pediatric ward were included in the study.

### Sample size determination and procedure/technique

The sample size was determined using single population proportion formula using the incidence of first attempt peripheral intravenous cannulation failure of 50%, at 95% confidence interval and 80% power.



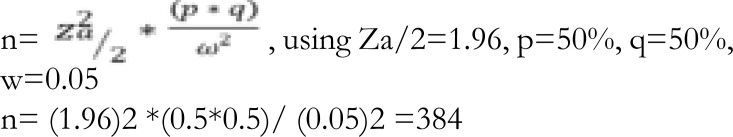



Then, by adding 10% non-response rate (384+38), the final calculated sample size for this study was 422. Then, the total sample size was allocated for each unit (paediatric ward, paediatric emergency, OR, and ETAT). Finally, the study participants were selected using a simple random sampling technique.

### Dependent variable

First attempt peripheral intravenous cannulation failure.

### Independent variables

Patient variables: Age, skin color, BMI, weight for age, existing medical condition.

### Clinician variables

Years of service, training, number of previous IV cannulations

### Equipment and procedure variables

Using vein viewer, cannula size, visibility using a tourniquet, insertion site, extremity selected.

### Operational definitions

#### Failure

Any attempted peripheral IV cannulation for any reason that has failed to function properly with /without flashback of blood at first trial.

#### Attempt

Each individual skin piercing with or without a flashback of blood. Redirecting the needle tip while underneath the skin would not be considered as a separate attempt.

#### Success

An attempt would be considered successful when the line could be flushed freely with saline without any signs of extravasation.

#### Under-nutrition

Expressed using weight for age for children with the age of < 5 years old.

### Data collection tool and procedure/technique

A structured and pre-tested interviewer-administered questionnaire was used to collect the data. The data collection tool was developed after reviewing different literatures 3, 7, 9–11, and it was prepared in English language. The tool contains patient variables (age, sex, anthropometric measurements), clinician variables (IV insertion experience, service year), and procedure variables (site of insertion, any assist device aid used, extremity selected, and size of cannula). The reliability of the tool was also established with a reliability coefficient (Cronbach's alpha score) which was 0.76 for patient variables, 0.79 for clinician variables, and 0.82 for procedure variables. Before data collection, training was given for data collectors and supervisors. The data collectors also have informed the parents about the aims/purposes, risks, and possible benefits of the study, the right and refusal to participate in the study, and the collected information would be kept confidential. After all, those parents who were willing were involved in the study.

The data were collected by direct observation and interviewer-administered questionnaire. Five senior staff nurses working at Debre Tabor referral hospital (three nurses at day and two nurses at night) shift were collecting the data. The outcome was recorded either failure (no) or success (yes). Time to success or failure in minutes at the first trial was recorded using a stopwatch. Minutes spent from the first application of the tourniquet to either intravenous line partly secured and flushed adequately or the nurse decided to abort the first attempt was the follow-up period.

Personal information of the patients and clinicians were collected after the procedure to avoid the interruption of care. But, the data about the procedure (site, side, size of cannula, and time spent) were collected by merely observation during the instant of the procedure. Similarly, other possible factors like shift and working day/weekend day were recorded during the follow-up period.

### Data quality control, processing and analysis

The pre-test was done to check the reliability of the data collection tool, then variables with Cronbach's alpha test <75% were removed. The training was given for data collectors and supervisors about the objective of the study, data collection tool, ways of data collection, checking the completeness of the tool, and how to maintain confidentiality. The collected data were checked for completeness, cleaned, edited, coded manually, and entered into Epi data version 4.2, and exported to Stata software version 14 for analysis. Double data entry was done for its validity and compared to the original data. Descriptive measures were presented using tables.

Whereas, summary measures were also presented using mean and median. Cumulative incidence was reported for first attempt peripheral intravenous cannulation failure rate. Besides, measures of variation were presented using standard deviation. Additionally, chi-square was also used to compare the variation between categorical variables. Binary logistic regression was used to select the candidate variables at p<0.25 for the final model and Crude Odds Ratio (COR) was computed for bivariate analysis. Then, multivariable binary logistic regression was used to remove confounding variables and compute Adjusted Odds Ratio (AOR). The final model of fitness was checked using Homer and Lemeshow test at p>0.05. Finally, the association was declared using AOR at a 95% confidence level and ≤0.05.

### Ethical consideration

Ethical clearance was obtained from Debre Tabor University, College of Health Science, Research and Community Service Committee. Consent to participate in the study was obtained from the parents or legal guardians by data collectors. Assent was also obtained from patients who were able to give assent. Any communication about data was using numerical codes. For clinicians who participated in the study, informed consent to participate in the study was signed and their privacy not to be watched upon doing the insertion was maintained.

## Results

### Socio-demographic Characteristics of children and clinicians

Of the total 422 respondents, 379 of them were included in the final analysis giving a response rate of 89.81%. Around 224 (59.10%) children were in the age of less than 2 years old. More than half the clinicians, 250 (65.96) were also in the age of 24–35 years old. The mean ages of the children and clinicians were 1.86 (SD±0.39) and 33.75 (SD±2.98) years old respectively. Nearly half,184 (48.68%) of the children were in subcritical disease conditions. Regarding the clinical experience of the clinicians, only 61 (16.09%) had no any previous experience in NICU/Pediatric ward/ETAT and around 170 (44.85%) had an experience of 1–5 years. More than three quarters ,323 (44.85) of the clinicians had educational status of BSc degree in nursing. More than 90% (355) of the clinicians had no any special training about IV cannulation. About 174 (45.91%) of clinicians also had 101–800 IV canulation exposures (Table 1).

**Table 1:** Socio-demographic and related characteristics of children and clinicians at Debre Tabor referral hospital,Northwest Etiopia,2020(n=379)

Variables	First success		
	No (n=110)	Yes (n=269)	Total (n= 379)
**Child age** < 2 Years 2 years and above	75 35	149 120	224 155
**Child sex** Female Male	32 78	128 141	160 219
**Clinician sex** Female Male	73 37	161 108	234 145
**Clinician age** < 24 24–35 >=35	6 72 32	8 178 83	14 250 115
**Severity of the disease** Critical Subcritical Que	53 49 7	106 135 28	159 184 35
**Work experience in years** < 1 years 1–5 years >5 years	33 62 15	92 108 69	125 170 84
**Educational status of the clinicians** Diploma Diploma Degree/BSC MSC	12 93 5	35 230 4	47 323 9
**Experience in NICU/ PW/ETAT** Yes No	98 12	220 49	318 61
**Training** Yes No	5 105	19 250	24 355
**Number of previous IV insertion** <100 101–800 >=801	7 66 37	45 108 166	52 174 153
**Vein visible with tourniquet** Yes No	78 32	246 23	324 55
**Site of insertion** Hand Leg Scalp	84 12 14	221 26 22	305 38 36
**Cannula size** 18 Gg 20 Gg 22 Gg 24 Gg	7 1 6 96	7 5 40 217	14 6 46 313
**Time spent** <5 minute 5–10 minute >10 minute	26 46 38	109 103 57	135 149 95
**Vein scope/trans illuminator used** Yes No	35 75	41 228	76 303
**Skin color** Black Dark White/red/fair	8 35 67	28 79 162	36 114 229
**Side of hand** Left Right	43 40	151 78	194 118
**Site of hand** Cubital fossa Fore arm Hand	5 4 74	4 44 181	9 48 255

### Incidence of first attempt peripheral intravenous cannulation failure

This study showed that the incidence of first attempt peripheral intravenous cannulation failure among children admitted to Debre Tabor referral hospital was found to be 34.83% (132) (95% CI: 30.18%-39.78%).

### Predictors of first attempt peripheral intravenous cannulation failure

Self-payment funding (AOR=2.74; 95% CI: 1.07–7.02), vein visibility with a tourniquet (AOR=0.17; 95% CI: 0.06–0.45), forearm site (AOR=0.08; 95% CI: 0.01–0.45), vein scope use (AOR=2.7; 95% CI: 1.31–5.54), and child age 24–59 months (AOR= 0.34; 95% CI: 0.12–0.93) were associated with first attempt peripheral intravenous cannulation failure among children admitted to Debre Tabor referral hospital (Table 2).

**Table 2:** Predictors of first attempt peripheral intravenous cannulation failure among children admitted to Debre Tabor referral hospital, Northwest Ethiopia, 2020(n=379)

Variable	Category	Failure	Success	COR (95% CI)	AOR (95% CI)
Child sex	Male	88	131	1.65(1.03,2.17)	1.34(0.77,2.32)
	Female	44	116	1	
Severity classification	Subcritical/ priority	64	120	1.04(0.82,1.32)	1.00(0.57,1.75)
	Que	8	27	1.06(0.56,3.88)	0.90(0.23,3.49)
	Sever/emergent	59	100	1	
Funding sources	Self-payment	80	122	2.87(1.36,6.57)	2.74(1.07,7.02)*
	Social health insurance	39	80	2.58(1.72,6.92)	2.38(0.89,6.35)
	Free service	13	45	1	
Previous experience in NICU/PW	Yes	14	47	2.01(1.09,4.54)	1.76(0.76,4.05)
	No	118	200	1	
Training on IV insertion	Yes	5	19	0.31(0.07,1.19)	0.26(0.05,1.25)
	No	127	228	1	
Vein visible with tourniquet	Yes	98	226	0.23(0.02,0.39)	0.17(0.06,0.45)***
	No	21	34	1	
Side of hand	Right	46	72	1.46(0.88,2.35)	1.41(0.81,2.48)
	Left	56	138	1	
Site of hand used	Fore arm	8	40	1.01(0.03,0.52)	0.08(0.01,0.45)**
	Hand	89	166	0.27(0.06,1.02)	0.21(0.04,1.05)
	Cubital fossa	5	4	1	
Vein scope used	Yes	38	38	3.4(1.43,5.67)	2.7(1.31,5.54)**
	No	94	209	1	
Child age	24–59 months	12	45	0.36(0.23,0.86)	0.34(0.12,0.93)*
	>=59 months	30	68	1.03(0.52,1.32)	0.84(0.44,1.60)
	< 24 months	90	134	1	

**Note:** *p<=0.05; **p<=0.01; ***p<=0.001; ETAT: Emergency Triage Assessment and Treatment; NICU: Neonatal Intensive Care Unit

## Discussion

The finding of this study revealed that the incidence of the first attempt peripheral intravenous cannulation failure rate among children admitted to Debere Tabor referral hospital was nearly 35% (132). Similarly, self-payment funding, vein visibility with a tourniquet, forearm site, vein scope use, and child age of 24–59 months old were independent predictors of first attempt peripheral intravenous cannulation failure.

This study showed that the first attempt peripheral intravenous cannulation failure rate was 34.83% which was in line with a rate reported in Bhutan (36%) 9; but it was lower than the study conducted in Netherland (55%) 4 and South-eastern United States 40% 12. However, the finding of this study was higher than the findings from Australia (27%) 13, Queensland (24.8%) 14, Brazil (10.5%) 3 and Turkey (24.7%) 15. This variation might be due to the presence/absence of training about IV cannulation, work experience of the clinicians, the presence/absence of assistive devices (like vein tourniquet), and nutritional status of children in the study settings.

Moreover, this study showed that self-payment funding has attributed to first attempt peripheral intravenous cannulation failure at a rate of 2.74%. It might be due to clinicians may give more emphasis for those children who were receiving the service/IV cannulation by their own payment as compared with those children who were receiving the service exemptedly. Additionally, this study indicated that visible vein with a tourniquet and site of hand(forearm) have 83% and 92% less risk of getting the first attempt peripheral intravenous cannulation failure respectively. These might be due to the fact that applying tourniquet increases the visibility of the vein and veins which are found in the forearm of the children are highly visible and easily accessible. Similarly, children with the age of 24–59 months old had 64% less risk of getting the first attempt peripheral intravenous cannulation failure. It might be due to the fact that children in the age of 24–59 months old have easily accessible vein as compared with children with the age of less than 24 months old. On the contrary, this study showed that using vein scope increases the risk of getting the first attempt peripheral intravenous cannulation failure rate by 2.7%. This might be due to the fact that the vein might be easily raptured and the first attempt of peripheral IV cannulation might be failed.

## Limitations of the study

Since the study was conducted in a single- center, it might be a limiting factor not to address multicentric settings with different characteristics of the study populations. This study might also be subjected to recall and social desirability biases.

## Conclusion

This study revealed that the incidence of the first attempt peripheral intravenous cannulation failure was high. Generally, this study also showed that self-payment funding, vein visibility with a tourniquet, forearm site, vein scope use, and child age of 24–59 months old were independent predictors of first attempt peripheral intravenous cannulation failure.

## Recommendations

**i.** The Ministry of health-Ethiopia, regional health bureau, zonal health department, and all the concerned bodies shall strengthen their contributions of giving sustainable trainings about nursing arts including IV cannulations for all clinicians working in the area and the country at large.

i**i.** Nurses also shall give more emphasis to choose appropriate sites(forearm) and use tourniquets; but they shall avoid using vein scope sites while attempting peripheral intravenous cannulations.

## Data Availability

All data used for the study were included in the manuscript.
